# “Algal-dromes”: a novel conceptual approach to illness in humans exposed to harmful algal bloom toxins

**DOI:** 10.3389/ftox.2026.1749427

**Published:** 2026-03-13

**Authors:** Brett Johnson, Mindy Richlen, Jeffrey Lai, Michael J. Twiner

**Affiliations:** 1 Department of Emergency Medicine, Maine Medical Center-Biddeford, Biddeford, ME, United States; 2 Biology Department, Woods Hole Oceanographic Institution, Woods Hole, MA, United States; 3 Division of Medical Toxicology, Department of Emergency Medicine, University of Massachusetts Chan Medical School, Worcester, MA, United States; 4 Department of Emergency Medicine, Wayne State University, Detroit, MI, United States; 5 Integrative Biosciences Center, Wayne State University, Detroit, MI, United States

**Keywords:** amnesic shellfish poisoning, ciguatera, cyanobacteria, diarrhetic shellfish poisoning, paralytic shellfish poisoning, red tide

## Abstract

Although adverse health effects from harmful algal bloom (HAB) toxins have been described since antiquity, the true worldwide incidence and disease burden has yet to be defined. This is in part due to lack of reporting and under-recognition of exposure. Furthermore, when human exposure does occur, there exists little consensus on optimal treatment strategies for specific HAB events or confirmatory laboratory testing capabilities. Current management strategies largely rely on primary prevention through public health efforts, often undertaken at the state and local level. As serious illness is rare, current management of acute algal poisoning is mostly based on expert opinion and case reporting. Despite this, known incidence of human exposure to HAB toxins is increasing due to shifting environmental conditions, rising global seafood demand, and growing population density and development along coastal and freshwater bodies. This rise in human exposures underscores the pressing public health need to address current knowledge gaps. This paper provides a comprehensive review of many of the major algal toxins (specifically ciguatoxin, saxitoxin, azaspiracid, brevetoxin, okadaic acid, dinophysistoxin, domoic acid, and cyanotoxins), the management strategies associated with suspected poisoning, and presents a novel term to describe the unique syndromes associated with their illness; “algal-dromes.”

## 1 Introduction

Dinoflagellates, diatoms, and cyanobacteria are aquatic microorganisms that have inhabited the Earth for millions of years in both marine and freshwater environments. Under certain conditions, several of these species can rapidly proliferate and produce toxins that cause illness in wildlife and humans. Collectively these events are known as “harmful algal blooms,” or HABs (Anderson et al., 2021). These HABs are globally distributed, with the occurrence of specific toxins and illness largely following regional patterns defined by endemic species. Due to international travel and the global trade of seafood, HAB-related illnesses are increasingly seen outside of endemic areas (Mattei et al., 2014). Additionally, climate change, warming oceans, and eutrophication are altering the geographic distribution and bloom dynamics of some endemic organisms and regionally specific toxin profiles (Gobler, 2020). Predictive models also show that changing environmental conditions may further increase bloom magnitude and the prevalence of toxins and HAB-related illnesses in some regions (Gobler, 2020). As a result, regardless of region, healthcare providers must maintain a high degree of suspicion when encountering patients presenting with illness after aquatic exposures including ingestion, inhalation and/or dermal. When bloom events occur, their impacts not only influence public health, but can also have social consequences to communities, and significant long-term repercussions on multiple economic sectors (Hoagland and Scatasta, 2006; NOAA, 2021).

Epidemiology related to HABs is largely based on reports to local and national health agencies. To improve data on the impacts of HABs to humans and animals in the US, the Centers for Disease Control and Prevention (CDC) launched the One Health Harmful Algal Bloom System (OHHABS) surveillance and reporting system, which encourages data reporting by state and territorial public health departments and their designated environmental health or animal health partners (CDC, 2025). Despite increasing bloom incidence, HAB-related illnesses remain underreported for several reasons: individuals with mild or self-limited symptoms may not seek medical attention; illness caused by HAB toxins may overlap with non-toxicologic etiologies; healthcare providers who lack experience with HAB-associated illness may not consider toxin exposures as the etiology for their patients’ symptoms; and rapid confirmatory testing may not be available even when exposure to HAB toxins is suspected (Vigar et al., 2024). Currently, management strategies for HAB toxin poisonings are largely based on case reports and expert opinion.

With the rising incidence of HAB events, public health experts, healthcare providers, medical toxicologists, and poison centers will increasingly be called upon to manage illness and provide expertise in surveillance, mitigation, management, and testing. This paper provides a comprehensive review of the most common algal toxins, the syndromes associated with human illness, and proposed management strategies based upon the most current evidence and understanding of the mechanistic effects of these toxin groups. Furthermore, we formally present and describe the concept of algal-dromes as a novel way to illustrate the similar toxidromes across the various HAB toxin classes. Syndrome overlap likely reflects similarities among the chemical toxin structures and their mechanisms of action and routes of exposure.

## 2 Algal toxins

Algal toxins are produced by prokaryotic and eukaryotic microalgae in both marine and freshwater aquatic systems, and can cause adverse health effects through oral, respiratory, or dermal exposure. The molecular structures of algal toxins are structurally and functionally diverse secondary metabolites derived from unique biosynthetic pathways (Pulido, 2016). The major structural groups have been described: tetrahydropurines (saxitoxin), secondary amines (domoic acid, azaspiracid), macrocyclic imines (pinnatoxin, spirolide), linear and macrocyclic polyethers (okadaic acid, pectenotoxin, palytoxin), ladder-frame polyethers (brevetoxin, ciguatoxin) and cyclic peptides (microcystins) (Pulido, 2016; Pearson et al., 2010). Mechanisms of action and potency/efficacy of the individual molecules can be highly reliant on critical side groups or chemical moieties that have been elucidated via structure-activity relationship studies (Bouaïcha et al., 2019; Deng et al., 2025).

Based on their chemical properties and structures, algal toxins are broadly categorized as lipophilic or lipophobic, which influences their activity as well as uptake and transfer in food webs, target species, target host organ systems, route of exposure, pharmacologic mechanisms of action, and toxic effects. Research to date has largely focused on acute exposure, but subacute and chronic exposure are also likely harmful to human health and may contribute to malignancy. Other than various *in vivo* models, toxicokinetic data are scant and in most cases non-existent. Many HAB-causing algal species produce multiple toxins with varying potencies, not all of which are detected with current testing strategies. Furthermore, a single HAB species may be capable of producing neurotoxins, hepatotoxins, cardiotoxins, and gastrointestinal (GI) toxins. Information about the major algal toxin groups, causative organisms, routes of toxin exposure, and associated human poisoning syndromes is summarized in Table 1. With ongoing research on the subject, taxonomy of species and toxin nomenclature is subject to change and will likely expand.

**TABLE 1:** Summary of major algal toxin groups, associated human poisoning syndromes, routes of exposure, causative organisms, and proposed algal-drome.

Toxin group	Activity and structure	Causative organisms	Route of exposure	Human poisoning syndrome	Algal-drome^a^
Anatoxins (Merel et al., 2013; Plata-Calzado et al., 2022; Colas et al., 2021)	Bicyclic amine; mimics acetylcholine and acts as agonist at nAChR causing depolarization, naturally occurring organophosphate with capability of AChE inhibition	Benthic and planktonic freshwater cyanobacteria (primarily freshwater) *Dolichospermum*, *Cuspidothrix*, *Phormidium*, *Oscillatoria*, *Tychonema* and *Cylindrospermum*	Ingestion of contaminated water; inhalation of aerosols; dermal exposure	Cyanobacteria Poisoning -seizures, muscle paresthesias, weakness, respiratory paralysis -lacrimation, abdominal pain, nausea, vomiting, diarrhea	Ingestion 1°: Neurotoxic 2°: Gastrointestinal toxicity Topical 1°: Dermotoxic
Azaspiracids (AZAs) (Twiner et al., 2016; Twiner et al., 2008)	Polycyclic ether toxins; lipid soluble. Mechanism of action not well known; evidence of affinity and inhibition of the hERG potassium channels	Marine dinoflagellate *Azadinium spinosum*	Ingestion of bivalve shellfish (primarily mussels)	Azaspiracid Poisoning (AZP); -Symptoms are primarily gastrointestinal (nausea, vomiting, abdominal pain, and diarrhea); similar to DSP. -possible heart block and hypotension	Ingestion 1°: Gastrointestinal toxicity 2°: Cardiotoxic
Brevetoxins (BTX/PbTX) (Watkins et al., 2008; Diaz et al., 2019; Mattei et al., 1999; Fleming et al., 2011)	Ladder-frame polyether structure similar to CTX; lipid soluble. Bind to voltage-gated sodium channels causing persistent depolarization of target cells	Marine dinoflagellate; *Karenia brevis*	Ingestion of seafood (primarily bivalve shellfish); inhalation of aerosols	Neurotoxic Shellfish Poisoning (NSP) -Symptoms based on route of exposure -Ingestion: GI and neurological (stroke-like symptoms); some similarities to PbTx/NSP. -Inhalation: respiratory distress, bronchospasm, reactive airway	Ingestion 1°: Neurotoxic 2°: Gastrointestinal toxicity Inhalation 1°: Pulmonary toxicity
Ciguatoxins (CTXs): Pacific (P-CTX) Caribbean (C-CTX), and Indian Ocean (Friedman et al., 2017; Mattei et al., 1999)	Ladder-frame polyether structure similar to PbTX; lipid soluble. Bind to voltage-gated sodium channels causing neuro-excitability and persistent cell depolarization	Marine dinoflagellates; *Gambierdiscus* spp., *Fukuyoa* spp.	Ingestion of coral reef fish and sometimes bivalves from tropical/subtropical regions	Ciguatera Poisoning (CP); -Symptoms: nonspecific gastrointestinal (GI), neurological, and cardiovascular complaints; some similarities to PbTx/NSP.	Ingestion 1°: Neurotoxic (P-CTX > C-CTX) 2°: Cardiotoxic 3°: Gastrointestinal toxicity (C-CTX > P-CTX)
Cylindrospermopsins (CYN) (Pearson et al., 2010; Merel et al., 2013)	Stable tricyclic quinidine alkaloids; hepatotoxic and inhibits protein synthesis	Benthic and planktonic (primarily freshwater) cyanobacteria *Raphidiopsis, Dolichospermum, Microseira, Umezakia*	Ingestion of contaminated water or food; inhalation and dermal contact	Ingestion can cause hepatoenteritis-like illness: fever, headache, vomiting, bloody diarrhea, hepatomegaly and kidney damage with the loss of water, electrolytes and protein	Ingestion 1°: Gastrointestinal toxicity Topical 1°: Gastrointestinal toxicity 2°: Dermotoxic
Domoic Acid (DA) (Lefebvre and Robertson, 2010; Saeed et al., 2017; Pulido, 2008; Krasner et al., 2025)	Structural analog of kainic acid and glutamate; water soluble. Acts on ionotropic glutamate receptors, activating kainite and AMPA receptors. Activation causes an uncontrolled influx of calcium leading to neuroexcitation	Marine diatom; *Pseudo-nitzschia* spp.	Ingestion of seafood	Amnesic Shellfish Poisoning (ASP) -Symptoms include short-term memory loss, seizures, respiratory difficulty -Nausea, vomiting, diarrhea, abdominal pain	Ingestion 1°: Neurotoxic 2°: Gastrointestinal toxicity
Microcystins (MCs) (Pearson et al., 2010; Bouaïcha et al., 2019; FDA Human Foods Program, 2025; Arman and Clarke, 2021)	Toxic cyclic peptides; water soluble. Inhibit protein phosphatases (PP) 1 and 2A, enzymes crucial for cell regulation in tissues and organs, leading to disruption of cell signaling and oxidative stress	Benthic and planktonic freshwater cyanobacteria (primarily freshwater) *Microcystis, Dolichospermum, Fischerella, Gloeotrichia, Nodularia, Nostoc, Oscillatoria,* and *Planktothrix*	Ingestion, inhalation, dermal contact with contaminated water	Primarily gastrointestinal (nausea, vomiting, abdominal pain, and diarrhea), similar to DSP and AZP but primarily freshwater/estuary. May correlate with NALD, liver failure, DIC	Ingestion 1°: Gastrointestinal/hepatoxicity Inhalation 1°: Pulmonary toxicity Topical 1°:Dermotoxic
Okadaic Acid and Dinophysistoxin (DTX) (Twiner et al., 2016; Valdiglesias et al., 2013; Wang, 2008; Trainer et al., 2013)	Polyether toxins; lipophilic. Inhibit the enzyme activities of protein phosphatases, especially PP2A; disrupting intracellular processes and causing severe mucosal damages in the intestinal tract	Marine dinoflagellates; *Prorocentrum, Dinophysis*	Ingestion of seafood, primarily bivalves	Diarrhetic Shellfish Poisoning; Primarily gastrointestinal (nausea, vomiting, abdominal pain, and diarrhea), similar to AZP and MCs	Ingestion 1°: Gastrointestinal/hepatoxicity
Palytoxin (PTX) (De et al., 2010)	Complex and large polyketide toxins; structure includes lipophilic and water soluble regions. Target voltage-gated sodium and potassium channels	Marine dinoflagellates; *Ostreopsis*	Inhalation, ingestion, dermal exposure	PTX poisoning symptoms include dyspnea, cough, mucus production, gastrointestinal distress and neurological symptoms (paresthesia), bradycardia, renal failure, cyanosis, respiratory distress	Inhalation 1°: Pulmonary toxicity 2°: Gastrointestinal toxicity Topical 1°: Dermotoxic
Saxitoxins (STXs) (Pearson et al., 2010; Deng et al., 2025; Wiese et al., 2010)	Neurotoxic alkaloids that inhibit voltage-gated sodium channels. At least 57 STX analogs have been identified with various potencies. Can be hydrophilic or hydrophobic. Among the most potent natural neurotoxins known	Marine dinoflagellates; *Alexandrium catenella*; *Pyrodinium bahamense* Also produced by several cyanobacteria species (*Dolichospermum, Aphanizomenon*, *Raphidiopsis*, *Microseira*, *Planktothrix)*, but contribution to human exposure impacts in freshwater systems not well-known	Ingestion of seafood, primarily bivalves	Paralytic Shellfish Poisoning (PSP); gastrointestinal distress and neurological symptoms (including paresthesias, ataxia, and dysphagia). Severe cases may progress to respiratory failure	Ingestion 1°: Neurotoxic 2°: Gastrointestinal toxicity

^a^ Listed algal-dromes are prioritized based on proposed specificity and uniqueness of symptoms and/or clinical importance.

In general, suspected human exposure can be clinically ascertained based on patient history and predictable clinical presentations, aided by the concept of algal-dromes associated with specific algal toxins. In addition to symptomatic treatment, healthcare providers should consider the type and duration of exposure, as well as the number of individuals affected. Similar to most other toxicological presentations, confirmatory testing is often not available in a timely manner and should not delay acute clinical management. For suspected exposures involving ingestions, information about the food source, where it was obtained, amount consumed, preparation methods, and availability of leftovers for analysis may aid investigation. Matrices such as human serum, urine, stomach contents, or feces should be obtained as close to the exposure event as possible and may become helpful in confirmatory analysis. The majority of treatment strategies for algal toxin exposure involve supportive care and minimizing any further exposure. When applicable, consultation with regional poison control centers is recommended to help guide clinical care, document suspected exposure, and coordinate public health response efforts.

## 3 Ciguatoxin

### 3.1 Syndrome/incidence

Ciguatera Poisoning (CP) is caused by the consumption of coral reef fish contaminated with a complex array of neurotoxins collectively known as ciguatoxins (CTXs). These toxins are produced by tropical and subtropical dinoflagellate species in the genera *Gambierdiscus* and *Fukuyoa,* which inhabit macroalgae and detritus within coral reef regions (Cruz-Rivera and Villareal, 2006; Parsons et al., 2012). CTXs enter the coral reef food web via grazing by herbivorous fishes and invertebrates, and bioaccumulate in fish. Toxin levels are often highest in large, carnivorous reef fishes (e.g., grouper, snapper, king mackerel, barracuda, moray eel), many of which are targeted for human consumption. CTX has also been reported in invertebrates, including giant clam species in the South Pacific (Roué et al., 2016). Worldwide, CTX is found in tropical and subtropical latitudes and considered endemic in parts of the Caribbean, Hawai’i, and the South Pacific (Friedman et al., 2017). CP incidence is estimated at approximately 50,000 cases annually, but the true extent is hindered by misdiagnosis and under-reporting to health authorities (Lewis, 2001). Fatalities are rare. Although most CP outbreaks occur in endemic areas, globalization of seafood trade has resulted in illness from fish imported to temperate and/or landlocked locations (Gingold et al., 2014). In addition to outbreaks of food consumption, transmission has also occurred through breast milk (Blythe and Sylva, 1990; Graber et al., 2013).

### 3.2 Pathophysiology/molecular mechanism

Ciguatoxins are heat stable and include several different toxic polycyclic polyethers. At least 30 congeners comprise the Pacific ciguatoxins (P-CTX) and at least 12 congeners comprise the Caribbean variant (C-CTX). There is at least one known Indian ocean variant (I-CTX). CTXs are structurally similar to the lipid-soluble polyether brevetoxins produced by the marine dinoflagellate *Karenia brevis* (known to cause Neurotoxic Shellfish Poisoning or NSP; see below). CTXs bind to and activate voltage-gated sodium channels (VGSCs) causing neuro-excitability and persistent cell depolarization. Prolonged depolarization of susceptible sodium channels has been seen in skeletal muscle, heart, peripheral and central nervous system and is responsible for producing the known clinical symptoms (Watkins et al., 2008). Pacific-CTX is the most potent, producing clinical symptoms in humans at levels >0.08 ng P-CTX-1 g^-1^ fish flesh (Lehane and Lewis, 2000; Pasinszki et al., 2020). The LD_50_ of 0.25 ng P-CTX-1 g^-1^ in a murine intraperitoneal model establishes this congener as one of the more lethal known algal toxins (Nicholson and Lewis, 2006). Caribbean CTX (specifically C-CTX-1) is approximately ten-fold less potent than P-CTX-1 (Lehane and Lewis, 2000; Nicholson and Lewis, 2006). The U.S. FDA has proposed advisory levels of 0.10 ppb C-CTX-1 equivalent toxicity in fish from the tropical Atlantic, Gulf of Mexico, Caribbean, and 0.01 ppb P-CTX-1 equivalent toxicity in fish from Pacific regions (US FDA, 2022). In addition to CTXs, some species of *Gambierdiscus* and *Fukuyoa* have been shown to produce other polyether compounds such as maitotoxins (MTXs), gambierol, gambieric acids, and gambierone, but their contribution to CP is not established (Soliño and Costa, 2020).

### 3.3 Clinical symptoms and differential diagnosis

The ciguatera poisoning algal-drome typically presents with nonspecific GI, neurological, and cardiovascular complaints (Friedman et al., 2017). A prodrome of GI symptoms may include nausea, vomiting, abdominal pain, and diarrhea, typically within 6–12 h of food consumption. This may be followed by symptom resolution or may progress to more serious neurological and cardiovascular illness. Cardiovascular symptoms include bradycardia, heart block, and hemodynamic instability. Neurologic symptoms often appear within 1–2 days following onset of GI symptoms and may include numbness and tingling to extremities, headaches, sensation of loose teeth, nonspecific neuropsychiatric complaints, vertiginous symptoms, and muscle weakness. A sensation of hot and cold reversal, known as “cold allodynia” has also been reported and is described as a burning sensation when touching cold items (Pearn, 2001). This same phenomenon is also seen in some cases of NSP (Friedman et al., 2017). In severe cases of CP, paresthesias, severe dysesthesia, and hallucinations have been reported. Interestingly, there appears to be regional variability in symptoms likely related to the distinct variation in CTX congener exposures; C-CTX has been known to produce more GI symptoms, whereas P-CTX produces more neurological symptoms, and I-CTX has been documented to produce hallucinations (Hamilton et al., 2002). Post-exposure, there is also a phenomenon of symptom recurrence that has been shown to be triggered by several foods (particularly fish and other meats, nuts, alcohol), weight loss, and exercise (Friedman et al., 2017). Symptom recurrence is thought to be secondary to remobilization of lipophilic toxin stores and/or an immunologically-mediated sensitization (Gatti et al., 2021).

The differential diagnosis for CP is broad as there is much symptom overlap with other known metabolic, infectious, neurologic, and cardiovascular disease processes. GI symptoms may mirror viral or bacterial etiology, as well as mimic other food borne toxic exposure such as botulism, scombroid, shellfish poisoning, viral gastroenteritis, etc. Neurological symptoms may mimic acute ischemic events such as stroke, Guillain Barre syndrome, multiple sclerosis, as well as various meningitis and encephalitis etiologies. Cardiovascular symptoms often mimic other sodium channel activator pathology similar to known xenobiotic, plant alkaloids, animal/environmental toxins (seen in aconitine, grayanotoxin, *Veratrum* ssp.).

### 3.4 Management/treatment

#### 3.4.1 Supportive care

Consider administering activated charcoal in patients who are hemodynamically stable, have had recent ingestion, exhibit positive bowel sounds, are alert and oriented, and are able to protect their airway. Treat volume losses and hypotension with intravenous fluid (IVF) resuscitation. Manage electrolyte disturbances from GI losses with adequate repletion. Nausea and vomiting can be managed with antiemetics (use caution with QTc prolonging agents). Monitor patients showing signs of cardiac toxicity with telemetry. Serial electrocardiogram (ECG) monitoring can help detect changes in atrioventricular (AV) nodal blockade, QRS, and QTc intervals. Bradycardia should be treated with standard advanced cardiac life support (ACLS) protocols; atropine, mechanical or pharmacological pacing. Refractory hypotension is managed with intravenous vasopressors (Friedman et al., 2017). In rare cases of severe CP, patients may require intubation and mechanical ventilation for airway protection and respiratory support. Those with a diagnosis of CP should be counseled on risk of further exposure through consumption of carnivorous reef fish and symptom recurrence up to 6 months following illness.

#### 3.4.2 Antidotes/proposed specific treatments

There is no specific antidote for CP.

Initial analgesic treatment includes nonsteroidal anti-inflammatories (NSAIDs) and acetaminophen. Opioid analgesia may be necessary during acute illness. Long-term neuropathic pain associated with CP has been effectively treated with medications such as gabapentin, duloxetine, fluoxetine, amitriptyline, and pregabalin. Pruritus can be managed with antihistamines (Friedman et al., 2017).

Some studies support the use of IV mannitol (0.5–1 g/kg infused over 1 h) to help treat severe neurological symptoms. Mannitol’s pro-osmotic properties are thought to aid in reducing cerebral edema and neuronal swelling and believed to reduce neurological symptoms severity and duration (Mullins and Hoffman, 2017). Multiple doses of mannitol may be needed depending on disease severity and treatment response. When mannitol is considered, it is important to ensure adequate hemodynamic, intake/output, and electrolyte monitoring given mannitol’s profound pro-diuretic effects. Other studies have failed to show adequate benefit of mannitol when compared to IV saline placebo (Schnorf et al., 2002; Palafox et al., 1988; Mitchell, 2005).

#### 3.4.3 Testing options

To date, there is no consensus on appropriate human biomarkers for confirmatory diagnosis of CP. Exposures are usually confirmed by analyzing cooked and/or uncooked meal remnants (usually fish) using an *in vitro* cell (neuro 2a; N2a) assay to assess for composite toxicity and liquid chromatography-tandem mass spectrometry (LC–MS/MS) for structural confirmation (Abraham et al., 2012). Human cases have used post-mortem and serum matrices with high performance liquid chromatography (HPLC) and LC-MS/MS for confirmatory testing.

If CP is suspected, contact your regional poison center as well as your local and state department of public health for additional information on management, testing, and reporting. Additionally, the Food and Drug Administration (FDA) can receive and investigate reports via the instructions provided on their website (*see* Supplementary Material).

#### 3.4.4 Disposition

There is no defined observation period after a suspected CP exposure. Severe cases usually declare themselves within the first 12 h of symptom onset. Presence and duration of symptom severity should dictate disposition. Patients should be counseled on the potential for symptom reoccurrence up to 6 months after the initial exposure. Nursing mothers should be counseled on the risk of transmission through breast milk.

## 4 Saxitoxin

### 4.1 Syndrome/incidence

Paralytic shellfish poisoning (PSP) is a life-threatening human poisoning syndrome caused by the consumption of seafood, usually bivalves such as clams or mussels, that are contaminated with a group of neurotoxins that are collectively known as paralytic shellfish toxins, or PSTs. Of the PSTs, saxitoxins (STXs) are the most potent and bind to voltage-gated sodium channels in cell membranes, inhibiting sodium ion exchange (Deng et al., 2025). This disrupts normal cellular function and interrupts nerve transmissions, leading to paralysis. Saxitoxin was named after the butter clam (*Saxidomus)* from which it was first isolated. As SXTs are thermostable, cooking or freezing seafood does not eliminate the risk of illness. PSP-causing dinoflagellates produce other toxic compounds, such as spirolides and gymnodimines, although these have not been explicitly linked to PSP illness.

Like many other algal poisoning syndromes, the toxins that cause PSP are produced by bloom-forming dinoflagellates, most commonly species of *Alexandrium* and *Pyrodinium* and some cyanobacteria (Deng et al., 2025). *Alexandrium* is typically found in temperate and subarctic waters, while *Pyrodinium* is primarily found in tropical or subtropical waters. In marine aquatic systems, bivalve shellfish concentrate these toxins in their tissues during filter feeding, later posing a risk to human consumers. Although exceedingly rare, additional vectors can include puffer fish, crabs, and lobsters. Carnivorous fish that consume contaminated shellfish or crustaceans may also carry PSTs; however, toxins generally do not accumulate in large quantities in fish muscle which is the most commonly consumed meat. However, consuming whole fish products may increase exposure risk (Etheridge, 2010). This is also true with regards to the American lobster (*Homarus americanus*), where higher PST levels have been found in the hepato-pancreatic portion (e.g., “tomalley”) of the lobster that is not commonly consumed but is a delicacy by some.

Outbreaks of PSP are recorded annually throughout the world (Deng et al., 2025). Active surveillance programs monitor STX and dinoflagellate levels in shellfish meat and water columns, respectively. Local, state, and national regulatory bodies close recreational and commercial shellfish harvesting when levels reach thresholds set by the FDA and EU legislation. In the US, the level specified by the FDA is 80 μg STX eq. per 100 g shellfish meat.

### 4.2 Pathophysiology/molecular mechanism

There are over 40 structural congeners derived from STX, and toxicity depends on the type and quantity of congener produced. Among these, STX, neosaxitoxin, and gonyautoxin are the most potent, although individual variants can vary in toxicity by up to two orders of magnitude. STX and the various variants are heat stable, lipophilic, reversible inhibitors of VGSCs; similar to tetrodotoxin (Wiese et al., 2010) and opposite to CTX. Post-mortem analyses of PSP fatalities have shown detectable STX in gut contents, urine, bile, cerebral spinal fluid, liver, kidney, heart, lung, brain, spleen, adrenal glands, thyroid, and pancreas, suggesting a large volume of distribution. Metabolism of the toxin appears to be via hepatic glucuronidation (Garcia et al., 2010).

### 4.3 Clinical symptoms and differential diagnosis

Symptoms from PSP generally occur within 15–30 min of consuming contaminated food. Severe PSP exposure classically produces algal-drome symptoms within the nervous system; including perioral or finger digit numbness, loss of coordination, drowsiness, and nausea. Severe symptoms may include muscle paralysis and respiratory distress. Death may occur from diaphragmatic involvement and has been reported 2–24 h following ingestion (Etheridge, 2010).

Given the acute onset of symptoms, presentation may mimic other acute neurological emergencies such as hypoglycemia, acute ischemic events such as stroke, Guillain–Barré syndrome (GBS), multiple sclerosis, myasthenia gravis, meningitis, or encephalitis. Symptoms may also mimic other environmental or food borne exposures (infectious and toxic poisonings) such as arsenic, botulism, scombroid, tetrodotoxin exposure, and other algal-dromes (CP, amnesic shellfish poisoning (ASP) or domoic acid, neurotoxic shellfish poisoning (NSP) or brevetoxin).

### 4.4 Management/treatment

#### 4.4.1 Supportive care

Management of PSP is largely supportive. Aggressive IVF and respiratory support including intubation and mechanical ventilation may be needed (Twiner et al., 2012; Twiner et al., 2016). Consider activated charcoal in patients who present early with severe symptoms, who are alert and oriented, have active bowel sounds, and for which there is no concern for imminent airway compromise. Patients with severe symptoms require cardiac monitoring and serial ECG monitoring of QRS and QTc intervals (Deng et al., 2025).

#### 4.4.2 Antidotes/proposed specific treatments

There is no specific antidote for PSP.

Respiratory monitoring may include forced vital capacity (FVC) and/or negative inspiratory force (NIF) monitoring when concern for severe muscle weakness and respiratory component.

#### 4.4.3 Testing options

In many coastal regions where PSP recurs, long-standing surveillance programs operate to assist local resource managers and public health officials in preventing outbreaks. Analytical methods include screening mouse bioassays (MBA), with confirmatory testing using high-performance liquid chromatography and liquid chromatography–mass spectrometry (HPLC/LC-MS). Enzyme-linked immunosorbent assays (ELISA) are also employed, with confirmatory testing performed by HPLC. Case reports have documented successful detection of PSP toxins in human urine and plasma using ELISA and HPLC/LC-MS methods (Etheridge, 2010).

If PSP is suspected, contact your regional poison center as well as your local and state department of public health for additional guidance on management, testing, and reporting. Additionally, the Food and Drug Administration (FDA) accepts and investigates reports through the instructions provided on its website (See Supplementary Material).

#### 4.4.4 Disposition

There is no defined observation period for PSP. Severe cases usually declare themselves within the first 2–24 h of symptom onset. Presence and duration of symptom severity should dictate disposition.

## 5 Azaspiracid

### 5.1 Syndrome/incidence

Azaspiracid Shellfish Poisoning (AZP) is caused by the consumption of shellfish contaminated with azaspiracid toxin (AZA), which produced by the dinoflagellate *Azadinium* ssp. AZP was discovered in 1995 when eight people from the Netherlands developed gastrointestinal illness after eating mussels exported from Ireland. Their symptoms included nausea, vomiting, severe diarrhea, and abdominal pain. Since then there have been seven confirmed global poisoning events, all due to shellfish (mussels) originating from Ireland. AZAs have been reported in coastal waters and in shellfish from many regions of western Europe, northern Africa, South America, and North America as well as Japanese sponges and Scandinavian crabs. To date, there have been no reported deaths related to AZP (Twiner et al., 2008).

### 5.2 Pathophysiology/molecular mechanism

Azaspiracids are polycyclic ether toxins. They are lipophilic, colorless, odorless, and heat stable. There are over 20 known AZA analogues that have been identified in phytoplankton, crabs, and shellfish. *In vitro* models demonstrate AZA’s affinity for inhibition of the hERG potassium channels (Twiner et al., 2008). These potassium channels are important in the regulation of cardiac conduction, however it is unclear how or if this influences the human presentation of GI illness. In rodent models, AZA has been shown to cause cardiac PR prolongation on ECGs, arrhythmia, and a dose-dependent decrease in systolic and diastolic blood pressures (Twiner et al., 2012; Ferreiro et al., 2016). Rodent studies looking at smaller oral doses of AZA showed the development of intestinal hyperplasia and pulmonary tumors, suggesting carcinogenic potential in chronic exposure (Ito et al., 2002).

### 5.3 Clinical symptoms and differential diagnosis

Symptoms of AZP are predominantly self-limiting and gastrointestinal. Algal-drome symptoms include nausea, vomiting, abdominal pain, and diarrhea. Symptoms usually begin within a few hours after ingestion of AZA and can last up to 5 days. Illness may result in significant volume losses. The clinical presentation of AZP has significant overlap with other causes of acute gastrointestinal illnesses, including diarrhetic shellfish poisoning (DSP), viral gastroenteritis (e.g., norovirus), and bacterial gastroenteritis (e.g., *Vibrio*, *Salmonella*, *Escherichia coli*, *Shigella*).

### 5.4 Management/treatment

#### 5.4.1 Supportive care

Management of AZP is largely supportive and includes IVF administration, electrolyte repletion, and anti-emetics. Given the potential for QTc prolongation and arrhythmia, cardiac and ECG monitoring is recommended in severe cases (Twiner et al., 2008).

#### 5.4.2 Antidotes/proposed specific treatments

There is no specific antidote for AZP.

### 5.5 Testing options

Several authors have raised concerns about the poor sensitivity of the standard mouse bioassay for detecting AZA, compared with its traditional use for detecting PSP and DSP (Twiner et al., 2008). These traditional surveillance methods failed to prevent several European outbreaks of disease. Currently, HPLC screening tools are recommended. There is no standard for confirmatory testing of AZP.

When a foodborne source of AZP is suspected, contact your regional poison center and local and state department of public health for guidance on management and testing. Government organizations may also assist with sample collection and confirmatory analysis (See Supplementary Material).

### 5.6 Disposition

There is no defined observation period after AZP exposure. Presence and duration of symptom severity should dictate disposition.

## 6 Brevetoxin

### 6.1 Syndrome/incidence

Neurotoxic shellfish poisoning (NSP) is caused by the ingestion of brevetoxins (PbTx) produced by the dinoflagellate *K. brevis* (formerly *Gymnodinium brevis, Gymnodinium breve, and Ptychodiscus brevis*) that bioaccumulate in filter-feeding bivalves. Blooms of *K. brevis* are colloquially referred to as “red tide” and occur most frequently in the southeastern United States, particularly in the Gulf of Mexico (Anderson et al., 2021). Because *Karenia* cells lack cellulose thecae, they are easily ruptured by wave action, which causes aerosolization of brevetoxins that can be transported inland by wind. Inhalation of aerosolized brevetoxins has been linked to both acute and chronic respiratory ailments (Watkins et al., 2008), and studies have found that airborne brevetoxins can be transported two or more miles inland from coastal waters (Kirkpatrick et al., 2010). In regions most frequently impacted, *K. brevis* blooms are closely monitored to warn the public of potential exposure risks, including reports of respiratory irritation along beaches.

In addition to human health impacts, *K. brevis* blooms are associated with extensive ecological damage, and large bloom events have caused mass mortalities of fish, seabirds, manatees, dolphins, and turtles (Watkins et al., 2008; Fire et al., 2007).

### 6.2 Pathophysiology/molecular mechanism

There are over 30 known congeners of brevetoxin (PbTx). PbTx is a heat stable, colorless, lipid soluble polyether compound, with a structure and mechanism similar to CTX and opposite of STX. PbTxs bind to VGSCs causing persistent depolarization of target cells. In animal studies, PbTx had high bioavailability and large volume of distribution, with highest concentrations in the liver up to 5 days post exposure. PbTx is metabolized in the liver and excreted in bile and urine. It is hypothesized that ocean waves rupture *K. brevis* cells and aerosolize PbTx, which can be inhaled causing significant respiratory irritation. The mechanism for respiratory irritation is thought to be related to activation and opening of sodium channels on cholinergic neurons in the respiratory tract, stimulating smooth muscle contraction and bronchospasm (Spaulding, 2009).

### 6.3 Clinical symptoms and differential diagnosis

Symptoms of NSP algal-drome are directly related to the route of exposure. Following ingestion, symptoms can occur as early as 15 min after ingestion and include both gastrointestinal and neurological ailments (Watkins et al., 2008; Diaz et al., 2019). Common GI symptoms include nausea, vomiting, abdominal pain, and diarrhea. Neurological symptoms include headache, paresthesia, ataxia, limb paralysis, slurred speech, and miosis. Symptoms of PbTx inhalation occur almost immediately after exposure, and include cough, throat irritation, chest tightness, wheezing, and dyspnea (Diaz et al., 2019). Effects are likely worse in people with pulmonary disorders such as asthma or chronic obstructive pulmonary disease (COPD). Other reported symptoms from inhalation exposure include eye irritation and pruritus.

The neurologic manifestations of NSP can mimic other neurologic emergencies such as acute stroke, hypoglycemia, Guillain–Barré syndrome (GBS), or foodborne exposures such as botulism, PSP, ASP, or CP. Gastrointestinal symptoms can mimic other metabolic and infectious etiologies including DSP, viral gastroenteritis (e.g., norovirus), and bacterial gastroenteritis. Respiratory manifestations can also be the trigger for exacerbations of reactive airway diseases such as asthma or COPD.

### 6.4 Management/treatment

#### 6.4.1 Supportive

Treatment of NSP is largely supportive and includes IVF and electrolyte replacement. Activated charcoal can be considered in patients who present early after ingestion, with active bowel sounds, normal mentation, and no concern for imminent airway compromise. For cases of respiratory exposure, supportive measures may include inhaled bronchodilators (e.g., albuterol, ipratropium), steroids, and magnesium (Watkins et al., 2008; Diaz et al., 2019). Oxygen and positive pressure ventilation should be utilized as needed, and in the event of respiratory decompensation, the airway should be secured. Ventilator settings should consider permissive hypercapnia with a prolonged expiratory phase to avoid complications associated with increased positive end-expiratory pressure.

#### 6.4.2 Antidotes/proposed specific treatments

There is no specific antidote for NSP or aerosolized exposure to PbTx.

Given similarities in toxin structure and mechanism of action, some have suggested the use of IV mannitol as an adjunct therapy in cases of severe neurologic illness (Mattei et al., 1999). This remains controversial as there is no clinical data supporting its efficacy. If mannitol is considered, care should be taken to ensure adequate volume and electrolyte monitoring. Brevinal, a naturally occurring compound produced by *K*. *brevis*, was shown to inhibit brevetoxin in animal studies. Data in humans does not exist; however, this compound may have a potential role in the treatment of NSP as well as CP (Mattei et al., 1999; Gold et al., 2013).

Cardiac monitoring and serial ECG may be necessary if severe acute illness is suspected. Respiratory monitoring may include repeated peak flow measurements and FVC/NIF monitoring when there is concern for severe muscle weakness and respiratory compromise.

#### 6.4.3 Testing options

There is currently no consensus on the optimum confirmatory testing strategies for suspected NSP exposure. Human serum and plasma have been used to detect NSP using ELISA methods, and PbTx metabolites can be detected in human urine using LC-MS. Meal remnants can be analyzed using bioassays, ELISA, LC-MS and HPLC-MS/MC confirmatory testing methods.

When NSP exposure is suspected, contact your regional poison center and local and state department of public health for guidance on management and testing. Additionally, government organizations may aid in sample collection and confirmatory testing (see Supplementary Material).

#### 6.4.4 Disposition

Disposition is determined by the patient’s clinical presentation and response to supportive care. Patients with severe or refractory symptoms may require admission for further symptomatic control.

## 7 Okadaic acid and dinophysistoxin

### 7.1 Syndrome/incidence

Diarrhetic shellfish poisoning (DSP) results from ingestion of okadaic acid (OA) and dinophysistoxins (DTXs) produced by several species of dinoflagellates; notably *Dinophysis* and *Prorocentrum* (Reguera et al., 2012; Valdiglesias et al., 2013). These toxins can bioaccumulate in filter-feeding shellfish, posing a health risk after human consumption. In humans, DSP typically manifests as a self-limiting gastroenteritis generally consisting of nausea, vomiting, diarrhea, and abdominal pain (Wang, 2008).

Dinoflagellate species capable of producing DSP toxins are distributed globally. Outbreaks are often linked to the consumption of contaminated mussels, and have been reported from Europe, Asia, and North and South America. In the US, DSP is considered an emerging HAB concern; however, DTXs have been detected in shellfish along all coasts (Anderson et al., 2021). Given the self-limiting nature of the illness, DSP poisoning is likely underreported internationally.

### 7.2 Pathophysiology/molecular mechanism

Okadaic acid and dinophysistoxin are lipophilic macrocyclic and linear polyester compounds. They act by inhibiting specific serine and threonine residues of protein phosphatases (ser/thr protein phosphatase 1, 2A, and 5), which control secretion of sodium in intestinal cells (Twiner et al., 2016). This is a similar mechanism of action as microcystins (MCs) and results in gastrointestinal symptoms in humans. These protein phosphatases are also implicated in many cell processes including cell division, with a potential link to carcinogenesis when disrupted.

### 7.3 Clinical symptoms and differential diagnosis

Symptoms of DSP usually begin within 30 min of ingestion of food containing toxins and include nausea, vomiting, abdominal pain, and diarrhea (Wang, 2008). Algal-drome symptoms resolve within 2–3 days. Much like AZP and MCs, there is significant overlap with other infectious acute gastrointestinal illness including viral gastroenteritis, and bacterial gastroenteritis. Chronic exposure to OA and DTX may promote tumor formation in the gastrointestinal system (Valdiglesias et al., 2013).

### 7.4 Management/treatment

#### 7.4.1 Supportive

Treatment of DSP is supportive and includes intravenous fluid and electrolyte repletion for gastrointestinal losses. Antiemetics may be used to alleviate gastrointestinal symptoms.

#### 7.4.2 Antidotes/proposed specific treatments

There is no antidote for DSP.

#### 7.4.3 Testing options

Protein phosphatase inhibition assays and HPLC have been used to test food remnants and shellfish meat matrices.

#### 7.4.4 Disposition

There is no defined observation period for DSP. Presence and duration of symptom severity should dictate disposition, but symptoms should be self-limiting.

## 8 Domoic acid

### 8.1 Syndrome/incidence

Domoic Acid (DA) is the neurotoxin responsible for amnesic shellfish poisoning (ASP), a syndrome characterized by short-term memory loss, gastrointestinal distress, and neurological symptoms including headaches, confusion, weakness, seizures (Todd, 1993). Severe cases have resulted in death. ASP was first identified following a 1987 outbreak on Prince Edward Island, Canada, when consumption of contaminated mussels resulted in 107 documented cases and four fatalities (Bates et al., 1989). DA is produced by certain species in the diatom genus *Pseudo-nitzschia.* The toxin can accumulate in filter-feeding shellfish, finfish, and zooplankton, and impacts the nervous system of human and wildlife consumers. DA contamination has been documented in fish, crabs, and shellfish throughout Europe, Australia, and North and South America. In the US, its effects are widespread in marine birds and mammals, particularly on the West Coast, where recurrent *Pseudo-nitzschia* blooms are responsible for sickening and killing mammals and seabirds on a near annual basis (Lefebvre et al., 1999; Lefebvre and Robertson, 2010). Famously, Alfred Hitchcock’s *The Birds* is believed to be inspired by a DA poisoning event in California in 1961, when hundreds of strangely behaving and dying seabirds were observed in a coastal community. At the time the cause was unknown, but later analyses discovered that toxic *Pseudo-nitzschia* were present in high concentrations during the time of the event, implicating DA as the likely cause (Bargu et al., 2012).

### 8.2 Pathophysiology/molecular mechanism

DA structurally resembles the naturally occurring excitatory neurotransmitters kainic acid and glutamate, and acts on ionotropic glutamate receptors; activating kainate and α-amino-3-hydroxy-5-methyl-4-isoxazolepropionic acid (AMPA) receptors. Activation causes an uncontrolled influx of calcium leading to neuroexcitation (Saeed et al., 2017; Xi et al., 1997). Uncontrolled activation can cause irreversible damage to the hippocampus. DA is water soluble and a heat stable toxin. Rat models have shown maternal to fetal transfer of DA *in utero* as well as vertical transfer through breastfeeding (Maucher and Ramsdell, 2005; Maucher and Ramsdell, 2007).

### 8.3 Clinical symptoms and differential diagnosis

Early symptoms of ASP algal-drome include nausea, vomiting, diarrhea, and abdominal cramping. Exposure to higher levels of DA can lead to delayed symptoms up to 24 h post-ingestion, and include headaches, dizziness, hallucinations, confusion, permanent short-term memory loss, respiratory difficulty, tremors, cardiac arrhythmia, seizures, coma, and death (Pulid and o, 2008). ASP’s name comes from the rather unique documented experience of an antegrade memory disorder, in which affected individuals retained a number of cognitive abilities, but were noted to have delayed recall and cognitive complaints. Other documented symptoms include ophthalmoplegia, hemiparesis, and ataxia. In non-fatal case reports of ASP, most neurological symptoms self-resolved within 1–10 days.

The sequelae of long-term, low-level DA exposure is an area of active research but may result in a lower seizure threshold (Krasner et al., 2025). Given the acute onset of symptoms, presentation may mimic other acute emergencies such as acute ischemic events (e.g., stroke), seizures, hypoglycemia or other toxicologic exposures known to cause altered mental status and metabolic derangements (e.g., salicylate overdose).

### 8.4 Management/treatment

#### 8.4.1 Supportive

Treatment of suspected ASP is primarily supportive and should not be delayed for confirmatory testing. Gastrointestinal symptoms should be treated with IV fluids, antiemetics, and electrolyte repletion.

#### 8.4.2 Antidotes/proposed specific treatments

There is no specific antidote for ASP.

Seizures should be treated with benzodiazepines or phenobarbitol. Seizures refractory to benzodiazepines should be managed with propofol (Saeed et al., 2017; Krasner et al., 2025). Animal studies looking at a role for valproic acid with and without pyridoxine (vitamin B6) as a means to activate glutamate decarboxylase and convert glutamate to gamma-aminobutyric acid (GABA) have shown some promise in decreasing DA levels. In sea lions, alpha-lipoic acid has been used to mitigate seizures (Field et al., 2021). In cases of early human ingestions, there is likely a role for decontamination with activated charcoal. However, the administration of activated charcoal should be considered on a case-by-case basis given the risk of altered mental status and seizure.

#### 8.4.3 Testing options

Much like PSP, a mouse bioassay has been used for decades as a means of primary prevention and surveillance among shellfish. More recently, more specific testing using HPLC has been utilized. Surveillance testing includes monitoring phytoplankton levels and mussel/crab/clam tissue matrices to determine when shellfish areas need to be closed for harvesting. The FDA has adopted a level of 20 µg DA/g as the threshold for shellfish bed closure but close monitoring in rural areas is problematic. HPLC has been used for confirmatory testing in sea lions from a number of different matrices including aqueous humor, breast milk, stomach contents, feces, urine, and serum (Krasner et al., 2025).

When ASP and DA exposure is suspected in humans, notify your regional poison control center, as well as local and state authorities to assist with sample collection and confirmatory testing.

#### 8.4.4 Disposition

Given the potential for decompensation, those with objective neurological findings or intractable GI illness in the setting of suspected ASP/DA exposure should be admitted for further monitoring and symptomatic management.

## 9 Cyanotoxins (microcystin, cylindrospermopsin, anatoxin, nodularin)

### 9.1 Syndrome/incidence

Cyanobacteria, formerly referred to as “blue-green algae,” are diverse photosynthetic prokaryotes found in hot water vents, hot springs, freshwater, saltwater, deserts, and tundra climates worldwide. They are capable of being aerosolized and carried with weather and deposited in new areas. Cyanobacteria are some of the oldest known organisms on our planet. Some cyanobacteria are capable of producing toxins known to cause illness in humans, domesticated animals, and wildlife. These toxins include but are not limited to microcystins (MCs), nodularins, anatoxins, saxitoxins, and cylindrospermopsin (Merel et al., 2013).

Globally, freshwater cyanobacterial bloom events are increasing in frequency, and often occur in nutrient-rich, low-turbulence waters. Bloom events have been responsible for creating anoxic conditions that have led to fish kills. Cyanotoxins pose health risks via recreational exposure as well as consumption of contaminated drinking water. Unregulated dietary supplements may be another source of exposure (FDA Human Foods P rogram). Notably, a MC outbreak in 2014 in the water supply for the city of Toledo, USA resulted in an estimated 100 hospital visits for gastrointestinal complaints linked to contaminated drinking water from a bloom event in nearby Lake Erie. No deaths were reported; however, more than 500,000 residents were placed under a public health advisory that included bans on both drinking and showering with tap water, leaving a lasting economic and psychological toll on the community (Steffen et al., 2017). Another severe incident occurred in 1996 in Caruaru, Brazil, where dialysate contaminated with MC was linked to 26 deaths (Jochimsen et al., 1998).

Annually, cyanotoxin exposure results in significant mortality among wildlife, livestock, and domestic animals, usually due to contaminated drinking water sources (CDC, 2025). In North America, cyanobacteria exposures represent the most frequently reported HAB exposures, with freshwater MC exposure being most common. Marine estuaries connected to freshwater sources are yet another route of exposure, and cyanotoxins have been documented in shellfish and marine mammals.

### 9.2 Pathophysiology/molecular mechanism

Microcystins are the most widely studied cyanotoxin with over 90 known congeners. MC is a heat-stable cyclic heptapeptide. Through drinking water or food sources, ingested toxins are likely absorbed through organic anion transporting polypeptides (OAT-P) family transporters and inhibit serine and threonine protein phosphatases (ser/thr PP1 and PP2A) similar to OA, DTX, and nodularin. In the liver, this may disrupt endonucleases leading to DNA damage and liver failure (Melaram et al., 2024). Similar to amatoxin in cyclopeptide mushroom poisonings, the cyclic structure of MCs confers some resistance to metabolism via endogenous peptidases. Additionally, like anatoxin, cytotoxicity may be secondary to inhibition of RNA polymerase, and both toxins likely exhibit enterohepatic recirculation (Arman and Clarke, 2021). Nodularins are also produced by cyanobacteria and share a similar structure and characteristic beta-amino side chain, 3*S*-amino-9*S*-methoxy-2*S*,6,8*S*-trimethyl-10-phenyldeca-4*E*,6*E*-dienoic acid (ADDA). This conserved moiety is thought to play a crucial role in hepatotoxicity and may contribute to the postulated effects of MCs on non-alcoholic liver disease (Zhang et al., 2015).

Cylindrospermopsins (CYNs) are stable tricyclic quinidine alkaloids also produced by cyanobacteria. They are also known to cause hepatic dysfunction, which is thought to be secondary to inhibition of glutathione synthesis and oxidative stress pathways, resulting in cell lysis.

Anatoxins are capable of causing seizures and paralysis through various neurologic pathways. Anatoxin-a mimics the neurotransmitter acetylcholine (ACh) and acts as an agonist at the neuromuscular junction, activating the nicotinic ACh receptor (nAChR) and causing depolarization (Plata-Calzado et al., 2022). Anatoxin-a(s) is a naturally occurring organophosphate and irreversibly inhibits ACh esterase resulting in a surplus of ACh in the synapse (Colas et al., 2021). Although this may represent a novel therapeutic target for known antidotes, to date, bradycardia and cholinergic crisis have not been documented in severe cyanobacteria exposures.

Nodularin toxins are produced by species of *Nodularia* and are structurally and mechanistically similar to MCs in that they inhibit ser/thr PPs. Nodularin toxins are regarded as potential hepatotoxins, however there is lack of exposure data and epidemiology studies (Melaram et al., 2024). Nodularin exposure may cause allergic symptoms, skin rashes, gastrointestinal illness, and liver injury (Wharton et al., 2019).

### 9.3 Clinical symptoms and differential diagnosis

In humans, ingestion exposure sources may include accidental consumption of water contaminated with high concentrations of toxins, ingestion of contaminated seafood, or consuming blue-green algae supplements (Pearson et al., 2010). Generally, early algal-drome symptoms of severe cyanotoxin exposure include visual disturbances, gastrointestinal distress (nausea, vomiting, diarrhea, abdominal pain), and muscle weakness. Later manifestations may include dizziness, drowsiness, ataxia, motor weakness, respiratory and muscular paralysis (Withana et al., 2025; Lad et al., 2022). Case reports suggest that high dose exposures lead to a gastrointestinal prodrome followed by liver failure as in the case of a hemodialysis clinic using contaminated water (Jochimsen et al., 1998). Ovotoxicity and disseminated intravascular coagulopathy (DIC) have also been reported in the veterinary literature.

Dermal exposure to cyanotoxins has been reported after skin contact with blooms and may lead to transient dermatitis (Dhillon and Parker, 2025). Inhalation exposures have resulted in upper respiratory irritation and include reports of cold-like symptoms, rhinitis, sore throat, and bronchospasm. Thus far, all inhalation exposures reported have been self-limiting. Chronic exposure to cyanotoxins is an area of active research, with some studies suggesting a possible connection between non-alcoholic fatty liver disease (Zhang et al., 2015) and malignancy (Lim et al., 2023). An additional neurotoxin called ß-methylamino-L-alanine (BMAA) has been proposed by some to link chronic cyanotoxin exposure to neurodegenerative disease (Caller et al., 2009). Although an area of continued research, the EPA issued a disclosure statement in 2017 concluding there did not exist enough evidence to support such claims.

### 9.4 Management/treatment

#### 9.4.1 Supportive

Treatment for suspected MC exposure includes IVF for volume loss, antiemetics, and electrolyte repletion. Workup for suspected MC exposure includes assessment of hepatic and renal function. Seizures from associated neurotoxin exposure should be managed with benzodiazepines. Respiratory paralysis should be managed with supportive airway and ventilation strategies.

#### 9.4.2 Antidotes/proposed specific treatments

There are no specific antidotes for cyanotoxin exposures.

In cases where severe MC exposure is suspected or confirmed, or when patients present with evidence of hepatic injury, there may be a role for N-acetylcystine (NAC) (Zhao et al., 2018). Further proposed management strategies derive from the veterinary literature, where MC exposure and severe illness occur more frequently among domestic animals. Oral cholestyramine and silibinin have been used successfully in dogs, and in extreme cases, hemodialysis has been reported to be beneficial (Rankin et al., 2013).

#### 9.4.3 Testing options

There is no consensus on specific confirmatory testing pathways for suspected animal or human exposure. Research and case reports have described the use of HPLC on serum, plasma, feces, and urine, as well as post-mortem testing of brain, spleen, liver, and kidney.

Currently, drinking water and recreational water quality limit recommendations are set by the Environmental Protection Agency, however considerable state and local variability exists among specific water closure limits (United States Environmental Protection Agency, 2019).

#### 9.4.4 Disposition

Disposition is guided by the severity of clinical presentation and response to supportive care. Individuals with severe or refractory symptoms may require admission for further symptomatic management. Patients with elevated transaminases or suspected severe exposures should be admitted for close monitoring.

## 10 Other notable algal toxins

Palytoxins (PTXs) are a group of potent marine toxins produced by certain dinoflagellates that can accumulate in shellfish and fish. PTXs bind Na^+^/K^+^-ATPase receptors, causing efflux of potassium ions and influx of sodium ions, and resulting in cell depolarization (De et al., 2010). Human poisoning has been documented from ingestion, inhalation, and dermal contact. Symptoms after ingestion include bitter metallic taste, headache, nausea, vomiting, bradycardia, muscle weakness, ataxia, gastrointestinal hemorrhage, arrhythmia, cardiac ischemia and death. Palytoxins are known to be potent vasoconstrictors and an intense burning pain has been reported following dermal exposure. Inhalation of aerosolized palytoxin has been known to cause respiratory distress. A large outbreak occurred in 2005 when a bloom off the coast of Italy caused over 200 individuals to seek medical care (Ciminiello et al., 2014). No specific antidote exists for palytoxin poisoning, and medical care is supportive. Species of *Ostreopsis* have been shown to make a potent toxin very similar in structure to PTX and can cause significant respiratory symptoms in exposed individuals (Berdalet et al., 2022).

Clupeotoxism is characterized by the sudden onset of bitter metallic taste, nausea, vomiting, and diarrhea, followed by clinical deterioration. In severe cases, seizure, coma, and death after consumption of herring, sardine, anchovies have been reported. In at least one documented event, later analysis confirmed palytoxin as the causative agent (Ciminiello et al., 2014). There is no known antidote and medical care is supportive.

Chelonitoxin poisoning is associated with the consumption of contaminated sea turtle meat and is thought to be caused by the bioaccumulation of chelonitoxin from algal sources. Fatalities have been reported in the South Pacific and Caribbean, including the death of an infant in Micronesia following vertical transmission through breastmilk (Pavlin et al., 2015).

## 11 Discussion

### 11.1 Harmful algal blooms and algal toxins

Harmful algal blooms (HABs) have been described for centuries. HABs and their associated algal toxins have been topics of active multi-disciplinary research for decades due to their devasting effects on aquatic environments, marine and freshwater aquatic life, negative impacts on economical and recreational use of water resources, and known and perceived impacts on human health (Brenckman et al., 2025). There is growing evidence that HABs and algal toxins are expanding in both frequency and impact, likely related to climate change and eutrophication (Griffith and Gobler, 2020) as well as intensified monitoring and perceived bloom impacts (Hallegraeff et al., 2021). Even as monitoring improves, events remain underreported due to lack of awareness, detection methods, and testing resources, especially in remote regions. Although in many regions there are extensive monitoring programs and agencies in place to help ensure the safe human consumption of seafood and aquatic resources, the globalization of export and import of food resources increases the risk of exposure of algal toxins to unsuspecting, non-endemic regions (Klontz et al., 2009). To add to the complexity of HABs and their associated toxins, there are dozens of known HAB species with variations in their specific biology, chemistry, genetic composition, and triggers for toxin production (Wang and Huang, 2025) that result in thousands of different chemical structures with variable levels of potency and efficacy (Deng et al., 2025; Du et al., 2019), many of which remain unknown. Ongoing surveillance measures and analytical techniques will most certainly continue to identify additional harmful species and their associated toxins.

### 11.2 Human intoxications of algal toxins

It is often reported that algal toxins are responsible for between 50,000 and 500,000 human intoxications per year (Wang, 2008), and depending on the specific algal toxin, acute intoxications may result in mortality rates as high as 15% (Guillotin and Delcourt, 2022). As such, there is great concern for human health impacts due to exposure to algal toxins, either acutely or chronically (Vilariño et al., 2018). Humans at highest risk for are likely those residing close to aquatic systems (both marine and freshwater) and those most dependent on the aquatic system for their food and/or occupation. Within this cohort, individuals at highest risk for illness are those at extremes of age, with chronic diseases (e.g., asthma, COPD, diabetes mellitus), the immunosuppression, or with comorbid hepatic or gastrointestinal disease (Lad et al., 2022) that may adversely affect adsorption, distribution, metabolism, and/or elimination of the given toxin.

Acute human intoxication events have been well documented for ciguatoxin (Chinain et al., 2021), saxitoxin (Deng et al., 2025), azaspiracid (Twiner et al., 2008), brevetoxin (Fleming et al., 2011), okadaic acid/dinophysistoxin (Trainer et al., 2013), domoic acid (Krasner et al., 2025), and cyanotoxins (Soydan and Erbas, 2022), particularly microcystins (Lad et al., 2022). As outlined throughout this review, the major routes of exposure are ingestion, inhalation, and dermal. However, iatrogenic exposures remain a serious risk, as one of the most lethal microcystin exposures was via contaminated dialysate at a hemodialysis clinic (Jochimsen et al., 1998).

For nearly all of the algal toxins, the most predominant route of exposure is via ingestion of contaminated seafood (e.g., fish or shellfish), drinking water, and direct consumption of concentrated algae, commonly as nutraceuticals (Withana et al., 2025). Toxicologic effects are determined both by distribution of an algal toxin to target tissues, and its affinity for particular receptors in those target tissues (mechanism of action). The agonistic or antagonistic effects of that interaction are what determines the downstream toxicological impacts whether it be neurological (e.g., domoic acid, brevetoxin, ciguatoxin, anatoxin), cardiac (e.g., ciguatoxin), gastrointestinal (e.g., azaspiracid, okadaic acid, dinophysistoxin), or hepatic (e.g., microcystin). With the exception of domoic acid (Krasner et al., 2025), all of the other algal toxin structures undergo significant metabolism in mammals (often by the addition of various hydrophilic side chains via oxidation and/or conjugation pathways) prior to elimination via the renal and/or hepatic systems (Withana et al., 2025). Shellfish and other vector organisms also contribute to the diverse profile of algal toxins via their own metabolic pathways, all of which contribute to the milieu of algal toxins that may be found in a particular food source. In one particular instance, five algal toxins were found in seawater, and eight different algal toxins were found in shellfish (Sibat et al., 2023).

Over the last 2 decades, inhalation exposure has become more recognized as a major source of exposure of algal toxins to humans (Vej et al., 2024) and is particularly impactful in people with pulmonary disorders such as asthma or COPD (Fleming et al., 2007). A few particular HAB taxa, in particular *Karenia*, *Ostreopsis* and *Microcystis*, are more susceptible to lysis and aerosolization of their algal toxins. Common traits of these species include that they are predominately surface phytoplankton (versus benthic) and have friable cell walls/membranes, making them more susceptible to rupture via wave action or other surface turbulence such as aeration systems (Rogers and Stanley, 2023). The corresponding toxin groups most associated with inhalation exposure include brevetoxins (Fleming et al., 2007), microcystins (Cheng et al., 2007), and *Ostreopsis* toxins (Berdalet et al., 2022). Although there is a paucity of information regarding symptomatic effects of exposure, aerosolized microcystins, aerosolized brevetoxins and *Ostreopsis* toxins predominantly cause cough, increased mucus production, and dyspnea, which are likely due to a caustic inflammatory response and bronchospasm (Berdalet et al., 2022; Milian et al., 2007). These respiratory symptoms are likely secondary to the small (<2.5 μm) aerosolized toxins getting into the lower airways including the alveoli (Rogers and Stanley, 2023). For aerosolized microcystin, the greatest concentration was found in the 0.44–2.5 μm particulate size fraction (Shi et al., 2023). For aerosolized brevetoxin, the median particulate matter diameter was larger (7–9 μm) with most of the deposition in the upper airway, however there was detectible toxin in the <2.5 μm size fraction (Fleming et al., 2007). Although very little is known about any systemic effects of aerosolized brevetoxin or microcystin, there is some speculation that aerosolized microcystin may be linked to neurodegenerative diseases (Morris et al., 2025). Furthermore, aerosolized microcystin may be a source of chronic, low dose exposures that may result in increased liver disease (Zhao et al., 2020) and/or malignancy (Dhillon and Parker, 2025; Gorham et al., 2020). Despite the potential for population-level exposures from aerosolized algal toxins, most studies are not definitive (Lim et al., 2023).

Dermal exposure to several algal toxins often results in a self-limiting rash, irritation, hypersensitivity, and/or atopic dermatitis (Dhillon and Parker, 2025). Of greatest concern are brevetoxin, cylindrospermopsin, microcystin, nodularin, saxitoxin, and lipopolysaccharide endotoxins (LPS; a component of bacterial cell walls also found in cyanobacteria) (Dhillon and Parker, 2025; Stewart et al., 2006). Although absorption of algal toxins across an intact dermal layer is generally perceived as minimal, some studies are concerning for systemic absorption possibly leading to cutaneous malignancy; particularly in people that may have repeated occupational exposure (Nielsen and Jiang, 2020).

Other exposure routes such as *in utero* or in breast milk may play a role in fetal or neonatal exposure (Shum et al., 2020; Rogers et al., 2007; Lefebvre et al., 2018). An observational study suggests microcystin exposure may result in lower human birth weights (Jones, 2019).

### 11.3 Mechanisms of action of algal toxins

Just as there are numerous HAB species with structurally distinct algal toxins, there is broad diversity in the mechanism by which these algal toxins elicit their toxicologic effects (Withana et al., 2025). The biologic targets are varied, and include voltage-gated sodium channels (Watkins et al., 2008; Nicholson and Lewis, 2006; Etheridge, 2010), glutamate receptors (Krasner et al., 2025), acetylcholine receptors (Colas et al., 2021), protein phosphatases (Twiner et al., 2016; Arman and Clarke, 2021), potassium channels (Twiner et al., 2012), and cytotoxins (Pearson et al., 2010).

The majority of algal toxins can be categorized as: neurotoxic (domoic acid, saxitoxin, brevetoxin, anatoxin), cardiotoxic (ciguatoxin), gastrointestinal/hepatotoxic (microcystin, azaspiracid, okadaic acid, dinophysistoxin, nodularin), pulmonary toxic (brevetoxin, microcystins, *Ostreopsis* toxin/palytoxin), dermatotoxic (microcystin, cylindospermopsin, brevetoxin). Characteristic effects at the cellular and organ level are determined by the route of exposure, chemical properties of the toxin molecules, affinity of the toxin for specific targets (e.g., receptors, membranes, enzymes), and the action of the toxin on those targets.

### 11.4 Proposed concept of algal-dromes

**FIGURE 1:**
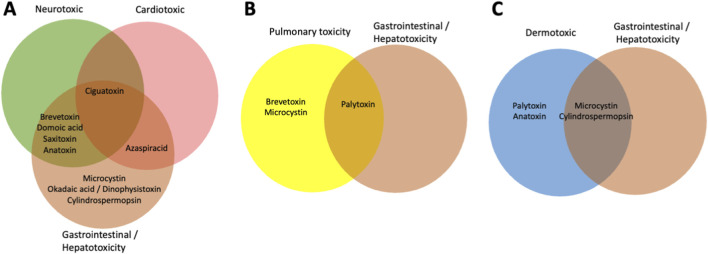
Conceptualized algal-dromes (based on specificity, uniqueness and clinical importance) for humans exposed to algal toxins via **(A)** ingestion, **(B)** inhalation, or **(C)** dermal.

One of the guiding principles of clinical toxicology is early recognition of potential patterns of toxin exposure based on patient history, symptoms, vital signs, and physical exam findings, commonly referred to as toxidromes. A toxidrome describes a constellation of signs and symptoms that occur with exposure to a group of similar substances, wherein common mechanism of action elicits a recognizable pattern that can help the clinician determine the likely underlying exposure (Nelson et al., 2019). A well-known clinical example of this is in opioid overdose, where the patient may present with altered mental status, respiratory depression, and pinpoint (miotic) pupils. Recognition of this syndrome guides healthcare providers to expeditiously provide treatment such as an antidote. Despite global human exposures to algal toxins approaching 500,000 people per year (Wang, 2008) as well as other marine mammals, livestock, and pets exposure, there is a paucity of information and guidance in the literature for healthcare providers to help with suspected identification and treatment of humans exposed to algal toxins. Thus, we propose the concept of algal-dromes where an algal-drome is set of clinically important signs and symptoms (e.g., neurotoxic, cardiotoxic, gastrointestinal/hepatotoxic, pulmonary toxic, and/or dermatotoxic) experienced by humans with suspected or confirmed exposure to algal toxins (Figure 1). Pattern recognition of signs and symptoms may allow for early recognition and treatment by clinicians. The proposed algal-dromes outlined in Table 1 have been categorized based on signs and symptoms unique to specific toxins, but not always the most observed (as many ingestions result in predominantly gastrointestinal symptoms). Systemic toxicity may vary based on route of exposure, dose, and potency of a specific toxin.

### 11.5 Overview of treatment strategies for humans exposed to algal toxins

As detailed above, the majority of treatment strategies involve supportive care and minimizing any further exposure. Consultation with your regional poison control/information center can not only aid in initial management but also resource coordination and is recommended in all cases of suspected algal toxin poisoning.

For the neurological algal-dromes, it is important to monitor the airway and provide appropriate airway support (including intubation as needed). Monitoring cardiac arrythmias and treatment of any electrolyte abnormalities is important. Pain control may be needed. Activated charcoal may be considered in patients who present early following an ingestion with severe symptoms. For suspected ciguatoxin (and maybe brevetoxin), mannitol should be considered. For suspected domoic acid, seizure precautions and treatment with anti-epileptics such as benzodiazepines or phenobarbital should be considered.

For cardiotoxic algal-dromes, cardiac monitoring and serial ECGs are essential. Treatment of any electrolyte abnormalities is important. If bradycardia or hypotension are present, consider atropine and/or vasopressors.

For gastrointestinal/hepatotoxic algal-dromes, it is important to consider dehydration and treatment of any electrolyte abnormalities. Symptomatic control with anti-emetics and histamine-2 or proton pump inhibitors may be helpful. Monitoring laboratory abnormalities is important including liver enzymes. For suspected severe microcystin exposure via ingestion, there is likely no harm in the provision of N-acetylcystine. Activated charcoal may be considered in patients who present early following an ingestion with severe symptoms.

For pulmonary toxic algal-domes, close monitoring of the airway is important and treatment with additional oxygen, beta-2 agonists, anticholinergics (via inhaler or nebulization), smooth muscle relaxers (e.g., intravenous magnesium), and/or steroids may be helpful. People with pulmonary disorders such as asthma or COPD are at high risk.

For dermatoxic agents, minimizing further exposure is extremely important by immediate decontamination with soap/water and the removal of any contaminated clothing. Systemic or topic anti-histamines and/or steroids may be helpful but symptoms are often self-resolving.

### 11.6 Summary and future directions

For decades, environmental and laboratory research involving global HAB species and algal toxin identification, detection, and mechanisms of action has provided high levels of safety for the public. To this end, research on the human health effects of HABs has been largely undertaken by public health, environmental science, and basic science disciplines (Pulido, 2016; Young et al., 2020) without extensive clinician involvement. There is growing recognition of the importance of engaging clinicians to improve the accurate diagnosis, treatment, and reporting of illness stemming from HAB toxin exposure, which in turn contributes to the development of baseline data on their occurrence and distribution (Carson et al., 2022). Regrettably, most North American medical school curricula do not include more than a simple mention of algal toxins (usually ciguatera) and healthcare providers generally lack the background in this particular area of study. Future research efforts should specifically seek to include education of healthcare providers for early identification of several algal-dromes, the expectant pathology, and critical treatments for these patients. One example includes the development of clinical training modules for healthcare providers (Association of State and Territorial Health Officials, 2022).

This review has several limitations. Most notably, most of the studies used for treatment recommendations are antidotal, case reports, small cohort studies, epidemiological and/or extrapolated from *in vitro* or *in vivo* studies, necessitating caution with application to the clinical treatment of humans. This manuscript was intended to include common known marine and freshwater algal toxins, but discussion does not include all known toxins, notably some more rare cyanotoxins. As further research identifies new organisms and toxins, this area is likely to expand. The concept of algal-dromes as a tool to assist healthcare providers in identifying predictable syndromes based on algal toxin exposure should help to inform early resuscitative care and aid in recognition of under reported events. These efforts are likely to improve health outcomes in the highest risk populations, such as communities dependent on subsistence aquaculture where public health surveillance infrastructure is limited. Specific priorities for future research include expanding diagnostic testing capacity, elucidating the effects of low-level and chronic HAB toxin exposure on human health, and assessing the influence of environmental change on disease incidence. Development of regionally tailored protocols may assist healthcare professionals in prompt diagnosis, effective management strategies, and improved risk communication during outbreak events.
