# Supplementary material for: “Algal-dromes”: a novel conceptual approach to illness in humans exposed to harmful algal bloom toxins
Source: Front Toxicol. 2026 Mar 13;8:1749427. doi: 10.3389/ftox.2026.1749427 (PMC13021141; doi:10.3389/ftox.2026.1749427)
Supplement: Supplementary file 1 [file Supplementaryfile1.docx]

Supplementary Material

# Appendix – Helpful Resources

ICD codes for reference

- T65.82 Toxic effect harmful algae and algae toxins
- Z77.121 Contact with and (suspected) exposure to harmful algae and algae toxins

CDC Resources

- Public Health Toolkit: <https://www.cdc.gov/harmful-algal-blooms/php/toolkit/index.html>
- CDC OHHABS reporting link: <https://www.cdc.gov/ohhabs/partcipants/index.html>
- Assessment tool: <https://www.cdc.gov/ohhabs/media/pdfs/OHHABS-Human-Case-Public-Health-Assessment-Tool-4.3.19-p.pdf>

FDA Resources

- **How to Report Seafood-Related Toxin and Scombrotoxin Fish Poisoning Illnesses:** <https://www.fda.gov/food/outbreaks-foodborne-illness/how-report-seafood-related-toxin-and-scombrotoxin-fish-poisoning-illnesses>
- **Food Remnants:** Any meal remnants should be immediately frozen and should not be eaten by any other individual(s). The meal remnants should be provided to health care providers or state/local public health authorities. The health care providers or state/local authorities will contact their local FDA District Office to coordinate submission of the remnants for analysis as deemed appropriate.
- **Fish/shellfish FDA Thresholds:** Institute of Medicine (US) and National Research Council (US) Committee on the Review of the Use of Scientific Criteria and Performance Standards for Safe Food. Scientific Criteria to Ensure Safe Food. Washington (DC): National Academies Press (US); 2003. Appendix C, Food and Drug Administration and Environmental Protection Agency Guidance Levels for Seafood. Available from: <https://www.ncbi.nlm.nih.gov/books/NBK221561/>
- **List of regional and local reporting numbers:** <https://www.cdc.gov/public-health-gateway/php/communications-resources/accredited-health-departments.html>
